# In Vitro Screening of Cattle-Derived Lactic Acid Bacteria Fermented Raw Milk for Probiotic Properties and Fecal Fermentation: Preliminary Insights into Potential Mechanisms Against Calf Diarrhea

**DOI:** 10.3390/ani16213464

**Published:** 2026-11-02

**Authors:** Lianguang Jiang, Meiqi Wang, Junzhao Xu, Huaxin Niu, Jingfeng Yang

**Affiliations:** College of Animal Science and Technology, Inner Mongolia Minzu University, Tongliao 028000, China; 13403443375@163.com (L.J.);

**Keywords:** cattle-derived lactic acid bacteria, fermented raw milk, in vitro fecal fermentation, short-chain fatty acids, calf diarrhea, gut microbiota

## Abstract

In this study, three bovine-derived lactic acid bacteria (LAB) strains were isolated from feces of healthy Simmental calves: *Lacticaseibacillus paracasei* (Lp), *Enterococcus hirae* (Eh), and *Enterococcus faecalis* (Ef). Bacterial suspensions were inoculated into pasteurized milk at a 2% inoculation ratio and anaerobically fermented at 37 °C for 24 h to obtain three kinds of LAB-fermented milks: *Lacticaseibacillus paracasei* fermented milk (LpM), *Enterococcus hirae* fermented milk (EhM), and *Enterococcus faecalis* fermented milk (EfM). Their probiotic potential was comprehensively evaluated through a series of in vitro assays, and in vitro colonic anaerobic fermentation was conducted using fecal inoculum from four Simmental calves (three biological replicates per group) to investigate the regulatory effects of these strains on the colonic microbiota and short-chain fatty acids (SCFAs). The three strains exhibited similar growth trends during raw milk fermentation. Compared with Eh and Ef, Lp showed significantly greater utilization of galactooligosaccharides (GOS) and lactooligosaccharides (LOS) (*p* < 0.01), whereas no significant difference in proteolytic activity was observed among the three strains (*p* = 0.192). LpM exhibited significantly higher water-holding capacity and cell-surface hydrophobicity than EhM and EfM (*p* < 0.01), along with a higher auto-aggregation rate (*p* < 0.01). Conversely, Eh and Ef displayed stronger co-aggregation with *Staphylococcus aureus*, *Escherichia coli*, and *Salmonella*, as well as greater antibacterial activity, with Ef being the most effective (*p* < 0.01). All three strains were highly sensitive to kanamycin, streptomycin, tetracycline, and doxycycline, whereas Eh and Ef showed intermediate sensitivity or resistance to several antibiotics. After fermentation, LpM and EhM retained IgG more effectively, while EfM consumed the least lactose. EhM produced the most acetic acid, LpM was dominant in propionic and butyric acid production, and both LpM and EhM generated more lactic acid than EfM (*p* < 0.01). The fermented milks displayed distinct 2,2-Diphenyl-1-picrylhydrazyl(DPPH) and hydroxyl-radical-scavenging capacities; LpM possessed the strongest antioxidant activity, followed by EhM, while EfM showed the weakest antioxidant performance (*p* < 0.01). During in vitro fermentation, both LpM and EhM produced more SCFAs than EfM. Through 16S rRNA gene sequencing it was revealed that LpM increased the abundance of *Butyricicoccus pullicaecorum*, *Lactococcus*, *Streptococcaceae*, and *Lachnospiraceae*. Although Eh and Ef could enrich genera such as *Bacillus* to inhibit diarrhea-associated pathogens, they also coincided with an increase in opportunistic pathogens including *Escherichia-Shigella*, *Klebsiella*, and *Enterococcus*. Therefore, Lp represents a promising candidate strain of bovine-derived lactic acid bacteria for fermented milk aimed at preventing calf diarrhea. Notably, Eh and Ef also enriched opportunistic pathogens, and further safety assessment is required before practical application.

## Simple Summary

Neonatal calf diarrhea remains one of the major challenges restricting the development of cattle farming, and probiotics are promising strategies to mitigate this problem. In this study, three bovine-origin lactic acid bacteria (LAB) strains were screened. Using an in vitro colonic fermentation model with fecal microbiota collected from healthy calves, we explored how fermented milk prepared by these strains regulates gut microbiota and short-chain fatty acid (SCFAs) production. Strain Lp showed good probiotic potential: its fermented milk could optimize intestinal microbial composition and boost the production of SCFAs, which is beneficial for gut barrier maintenance and may help prevent calf diarrhea. However, strains Eh and Ef also increased the relative abundance of opportunistic pathogens in the in vitro system, meaning their safety must be carefully assessed before field application. This study only provides in vitro preliminary evidence. Further in vivo animal tests are required to verify the actual effect of these fermented milk products.

## 1. Introduction

Calf diarrhea is a prevalent global disease with incidence and mortality rates of 55% and 15%, severely restricting breeding economic benefits. It mainly arises from infectious and non-infectious stimuli that disrupt intestinal barrier integrity. Long-term milk feeding is commonly applied to mitigate calf diarrhea, as milk enriches intestinal *Bifidobacterium* to block pathogen adhesion and enhances digestive brush-border enzyme activity to preserve intestinal physical barrier function [1,2]. Nevertheless, partial degradation and loss of milk nutrients during gastrointestinal transit limits its protective efficacy; Lactic acid bacteria (LAB) are effective in improving calf intestinal health by optimizing intestinal microflora structure, lowering fecal scores, and regulating intestinal immune barrier to relieve diarrhea [3,4]. Previous studies have verified that feces-derived *Lactobacillus plantarum*, *Lacticaseibacillus paracasei* (Lp), and *Limosilactobacillus reuteri* exert antibacterial, flora-regulating and short-chain fatty acid (SCFAs)-modulating effects [5,6,7]. However, the acidic and inflammatory intestinal microenvironment during diarrhea impairs LAB adhesion and barrier translocation, weakening their persistent probiotic effects. Fermented milk inoculated with probiotic LAB has emerged as a promising strategy for diarrhea prevention. Existing studies have confirmed that *Limosilactobacillus reuteri*-fermented milk can down-regulate virulence-associated genes of calf-diarrhea-relevant pathogens including *Salmonella Dublin*, *Escherichia coli* F5, and *Clostridium perfringens*, alleviate intestinal villus damage and inflammatory infiltration, and upregulate tight junction protein expression to repair intestinal barrier injury [8,9].

Cattle feces are a valuable reservoir of probiotic LAB. Among these candidates, both Lp and *Enterococcus* spp. are probiotics with broad prospects for production and application; however, only *Enterococcus lactis* and *Enterococcus faecalis* (Ef) have been certified as feed microorganisms in China. The probiotic germplasm resources of *Enterococcus* remain to be further developed. Lp is a widely used probiotic strain that can alleviate diarrhea by increasing the relative abundance of *Adlercreutzia* and *Veillonella* in the intestine, producing acetic acid and propionic acid, and regulating immunity [10]. Chandwe et al. [11] found that Lp significantly downregulated flagellin-induced inflammatory responses in animal rumen epithelial cells. *Enterococcus hirae* (Eh) can play roles in regulating intestinal flora [12]. Xi et al. [13] found that porcine-derived *E. hirae ZJ02* could efficiently utilize galactose, and this rapid galactose-utilizing capacity could improve fermentation quality, owing to its abundant glycoside hydrolase and glycosyltransferase genes. Ren et al. [14,15] discovered through in vitro culture that milk-derived Ef could selectively utilize 2′-fucosyllactose from human milk oligosaccharides and exhibited broad-spectrum inhibition against pathogenic bacteria such as *Escherichia coli* and *Salmonella* through bacteriocin production. Fermented milk produced by milk-derived *E. faecalis ZGBP5-53* can benefit animal health by generating bioactive peptides [16]. However, research on bovine-derived lactic acid bacteria fermented milk is currently scarce, and studies specifically examining the effects of bovine-derived lactic acid bacteria fermented milk on diarrhea in calves are even more limited.

We hypothesized that fermented milks prepared from bovine-derived LAB could modulate SCFA profiles and intestinal microbiota during in vitro fecal fermentation, while strain-specific differences would lead to distinct beneficial or potential adverse effects. This study aimed to investigate the in vitro probiotic properties of fermented milks produced by different bovine-derived lactic acid bacteria (Lp, Eh, Ef), demonstrating that bovine-derived LAB can leverage the advantages of milk to improve product quality, further regulate intestinal microbiota to alleviate calf diarrhea, and provide theoretical basis for the subsequent development and application of microecological preparations of bovine-derived LAB.

## 2. Materials and Methods

### 2.1. Milk Preparation, and Activation and Cultivation of Strains

This study employed a single-factor completely randomized design. Milk samples were collected from mid-lactation Holstein cows (40 days postpartum), centrifuged (8000 r/min, 30 min) to remove milk fat, pasteurized (63 °C, 30 min), and cooled to 37 °C for subsequent use. Samples were sourced from Xinniu Technology Animal Husbandry Co., Ltd. (Horqin Left Rear Banner, Tongliao City, Inner Mongolia, China).

Bovine-derived LAB were prepared in-house by the laboratory [6]. The laboratory-preserved strains were revived and purified as follows: frozen stock tubes were retrieved, and the strains were streaked densely in a crisscross pattern on MRS solid medium, followed by incubation at 37 °C for 48 h. Single colonies were picked and inoculated into liquid medium, shaken to ensure thorough cell dispersion, and incubated at 37 °C for 18 h. The cultures were then subcultured at a 2% inoculum rate and incubated at 37 °C; after 2 successive passages, the strains were used for the experiment. Bacterial strains were activated and harvested by centrifugation at 6000 r/min. Genomic DNA was extracted using a bacterial DNA extraction kit. The 16S rRNA gene was amplified by PCR with primers 27F/1492R (50 μL system; 94 °C for 5 min, followed by 35 cycles of 94 °C for 30 s, 56.1 °C for 30 s, 72 °C for 30 s; final extension at 72 °C for 10 min, preserved at 4 °C). Purified amplicons were sequenced by Sangon Biotech Co., Ltd. (Shanghai, China). The sequences were blasted against the NCBI database. A total of 20 closely related strains were selected, and a phylogenetic tree was constructed in MEGA 6.0 using the neighbor-joining method with 1000 bootstrap replicates to evaluate tree topology reliability. A total of 3 bovine-derived lactic acid bacterial strains were identified as *Lacticaseibacillus paracasei* (Lp), *Enterococcus hirae* (Eh) and *Enterococcus faecalis* (Ef). The concentration of each strain was adjusted to 1 × 10^9^ CFU/mL by the plate count method and reserved for later use. The phylogenetic tree of the selected strains is shown in Figure 1.

### 2.2. Culture Media and Main Reagents

MRS broth, agar medium, LB medium, pepsin, trypsin, bovine bile salts, antibiotic susceptibility disks, and Oxford cups were all purchased from Tianjin Sikesai Biotechnology Co., Ltd. (Tianjin, China). Indicator strains, including *Escherichia coli*, *Staphylococcus aureus*, and *Salmonella*, were revived from the strain collection of the Laboratory of the Inner Mongolia Autonomous Region Beef Cattle Disease Prevention and Control Engineering Technology Research Center. Penicillin, streptomycin, gentamicin, kanamycin, ampicillin, and ofloxacin were all purchased from BKMAM Biotechnology Co., Ltd. (Changde, China).

### 2.3. Preparation of Fermented Milk with Different Bovine-Derived LAB

The 3 activated LAB strains (Lp, Ef, and Eh) were centrifuged (10,000× *g*, 4 °C, 5 min) to remove the supernatant. The bacterial pellets were washed 3 times with sterile physiological saline, and the concentration was gradually adjusted to *OD*_600_ = 0.8–1.0 by adding physiological saline to prepare LAB suspensions. The suspensions were inoculated into milk samples at a 2% inoculum rate, mixed thoroughly, and incubated in an anaerobic incubator at 37 °C for 24 h. The 3 types of bovine-derived LAB fermented milk were labeled as follows: *Lacticaseibacillus paracasei* fermented milk (LpM), *E. hirae* fermented milk (EhM), and *E. faecalis* fermented milk (EfM). The fermented milk samples were stored at 4 °C for later use.

Fermented milk was centrifuged (10,000 r/min, 30 min) to prepare fermented whey and stored at 4 °C. The whey was vacuum filtered through a 0.45 μm sterile membrane and centrifuged (12,000× *g*, 20 min). The pellet was washed by resuspending in sterile saline and centrifuging three times. The final LAB suspension was adjusted to *OD*_600_ = 0.8–1.0 with sterile saline and kept at 4 °C.

### 2.4. Viability and Acidification Assessment of LAB in Fermented Milk

Growth was monitored in raw milk, not MRS medium. During 24 h of incubation at a constant temperature, *OD*_600_ was recorded every 3 h to generate the LAB viability curves. Simultaneously, the pH of each fermented milk was measured using a portable pH meter at the same intervals to construct the acidification curves.

### 2.5. Assessment of Nutrient Utilization by Different Bovine-Derived LAB

Following the protocol as described by Wang et al. [17], the ability of different bovine-derived LAB to utilize lactose (LOS) or galactose (GOS) was evaluated. The glucose in MRS medium was replaced with an equivalent amount of LOS or GOS, respectively, and 1.5% cresol purple solution (5 g/L) was added, followed by sterilization and storage for later use. The growth of bovine-derived LAB in MRS medium with LOS or GOS as the carbon source was observed.

The proteolytic activity of different bovine-derived LAB was determined using the skim milk agar plate method as described by Zhan et al. [18]. A 5 μL aliquot of LAB suspension was spotted onto skim milk agar plates (100 g/L skim milk, 15 g/L agar) and incubated at 37 °C for 48 h. The formation of a hydrolysis zone indicated the proteolytic activity of the strain.

### 2.6. Probiotic Properties of LAB in the Fermented Milk

#### 2.6.1. Water Holding Capacity and Surface Hydrophobicity of LAB in the Fermented Milk

The water holding capacity of LAB in the fermented milk was measured following the reported method [19]. Briefly, 10 mL of fermented milk was centrifuged (3000× *g*, 4 °C, 30 min), the supernatant was discarded, and the remaining precipitate was weighed. Each sample was assayed in triplicate, and the water holding capacity was calculated using Equation (1).

The surface hydrophobicity of LAB in the fermented milk was determined according to the method described by Li [7]. The *OD*_600_ of the LAB fermented milk whey suspension was recorded as *A*_0_. Subsequently, 2 mL of the suspension was mixed with 1 mL xylene and incubated at 37 °C for 2 h. After removal of the organic phase, the *OD*_600_ was measured as *A*_2*h*_, and the surface hydrophobicity rate was calculated using Equation (2).

Water holding capacity of LAB (%) = [mass of precipitate (g)/mass of sample (g)] × 100%(1)

Surface hydrophobicity rate of LAB (%) = [(*A*_0_ − *A*_2*h*_)/*A*_0_] × 100% (2)

#### 2.6.2. Auto-Aggregation Ability of LAB and Co-Aggregation Ability of LAB with Pathogens in the Fermented Milk

The auto-aggregation ability of LAB was determined according to the method described by Joyce et al. [20]. The *OD*_600_ of the LAB fermented milk whey suspension was measured as *A*_1_, followed by incubation at 37 °C for 3 h, after which the *OD*_600_ was measured again as *A*_3*h*_. The auto-aggregation rate was calculated using Equation (3).

The co-aggregation ability of LAB with pathogens in the fermented milk was determined according to the method described by Song [21]. First, following the method described by Wang [6], the pathogenic strains (*Staphylococcus aureus*, *Escherichia coli*, and Salmonella) were cultured in LB medium at 37 °C for 24 h, and the concentration was adjusted to 1 × 10^8^ CFU/mL to prepare pathogen suspensions. Equal volumes of the fermented milk whey suspension and the pathogen suspension were mixed, and the *OD*_600_ was measured as *A*_2_. After standing at room temperature for 18 h, the *OD*_600_ was measured as *A*_18*h*_, and the co-aggregation rate was calculated using Equation (4).

Auto-aggregation rate of LAB (%) = [(*A*_1_ − *A*_3*h*_)/*A*_1_] × 100% (3)

Co-aggregation rate of LAB with pathogens (%) = [(*A*_2_ − *A*_18*h*_)/*A*_2_] × 100% (4)

### 2.7. Determination of Antibacterial Activity and Antibiotic Susceptibility

The antibacterial activity of bovine-derived LAB fermented milk was determined according to the reported method by Zeng et al. [22]. *Staphylococcus aureus*, *Escherichia coli*, and *Salmonella* were used as indicator strains. MRS semi-solid medium was poured into sterilized plates. After solidification, sterilized Oxford cups were placed in the center of the plates. The indicator strains (2%) were inoculated into the medium and spread onto the upper layer of the plates. After the MRS medium solidified, 200 μL of fermented milk whey suspension was added into the Oxford cups. The plates were then incubated upside down at 37 °C under anaerobic conditions for 24 h. The diameter of the inhibition zone was measured with a vernier caliper. The antibacterial ability of the fermented milk against the indicator pathogens was evaluated based on the size of the inhibition zone; a diameter of less than 5 mm indicated no antibacterial effect.

The antibiotic sensitivity of bovine-derived LAB fermented milk was determined according to the method described by Huang [23]. MRS semi-solid medium was poured into sterilized plates. After solidification, 100 μL of the fermented milk whey suspension to be tested was spread onto the upper layer of the plates. Antibiotic susceptibility disks of kanamycin, streptomycin, gentamicin, tetracycline, doxycycline, erythromycin, ampicillin, ofloxacin, and lincomycin were placed in the center of the plates. The plates were incubated upside down at 37 °C under anaerobic conditions for 24 h. The diameter of the zone of inhibition was measured with a vernier caliper. The antibiotic sensitivity of the fermented milk was evaluated based on the size of the zone. The evaluation criteria are shown in Table 1.

### 2.8. Analysis of Active Substances in Fermented Milks

The concentrations of IgG, acetic acid, propionic acid, and butyric acid in fermented milk from bovine-derived LAB were determined using ELISA kits purchased from Shanghai Enzyme-linked Biotechnology Co., Ltd. (Shanghai, China), while lactose and lactic acid were measured by microplate assay kits purchased from Jiangsu Aidisheng Biological Technology Co., Ltd. (Yancheng, China). The ELISA assay was performed following the manufacturer’s instructions. The limit of detection was 0.1 ng/mL. The intra-assay CV < 10% and inter-assay CV < 12%. The standard curve exhibited good linearity with R^2^ = 0.95. The relative change rate (RCR) of active substances was calculated according to Equation (5). A positive RCR value indicates an increase in the active substance content after fermentation, reflecting the biosynthetic effect of LAB fermentation; a negative value indicates a decrease in the active substance content after fermentation, reflecting the degradative effect of LAB fermentation.

RCR (%) = [(C_fermented_ − C _raw_)/C_raw_] × 100% (5)

C_fermented_ is the content of the active substance in the fermented milk,

C_raw_ is the initial content of the active substance in the raw milk.

### 2.9. Determination of Antioxidant Activity

In this experiment, control raw milk (CONM) served as the control group. The hydroxyl radical and DPPH scavenging capacities of the 3 LAB strains before and after fermentation (Lp, Eh, Ef, LpM, EhM, and EfM) were determined. The LAB suspension was subjected to cell disruption according to the method of Wang to prepare cell-free extracts [6]. The bacterial suspension was sonicated at 200 W and 20 Hz for 10 min (working 9 s, resting 12 s), followed by centrifugation (9500 r/min, 15 min). The supernatant was collected and reserved for later use.

The hydroxyl radical scavenging rate before and after fermentation was determined according to the reported method [6]. Briefly, 2.0 mL of the cell-free extract or fermented milk whey was mixed with 2.0 mL of 9.0 mmol/L FeSO_4_ solution and 1.0 mL of 8.8 mmol/L H_2_O_2_ solution. The mixture was shaken well, allowed to stand for 10 min, and then 2.0 mL of 9.0 mmol/L salicylic acid/ethanol solution was added. After shaking, the mixture was incubated in a water bath at 37 °C for 30 min, and the absorbance was measured at 510 nm. In the sample blank group, H_2_O_2_ was replaced with blank reagent; in the blank control group, the sample was replaced with sample solvent. Each sample was assayed in triplicate, and the average value was taken. The hydroxyl radical scavenging rate was calculated using Equation (6). The DPPH radical scavenging rate before and after fermentation was determined according to the reported method [6]. Briefly, 2.0 mL of the cell-free extract or fermented milk whey was mixed with 2.0 mL of 0.2 mmol/L DPPH/ethanol solution. The mixture was shaken well and reacted in the dark for 30 min. The absorbance was measured at 517 nm, with absolute ethanol used for zero adjustment. In the control group, DPPH solution was replaced with 2.0 mL of 95% ethanol; the blank group consisted of 2.0 mL of DPPH solution mixed with 2.0 mL of 95% ethanol. Each sample was assayed in triplicate, and the average value was taken. The DPPH scavenging rate was calculated using Equation (7).

Hydroxyl radical scavenging rate (%) = [1 − (*A_x_* − *A_x_*_0_)/*A*_0_] × 100% (6)

*A_x_*: Fe^2+^ + sample + H_2_O_2_ + salicylic acid;

*A_x_*_0_: Fe^2+^ + sample + distilled water + salicylic acid;

*A*_0_: Fe^2+^ + distilled water + H_2_O_2_ + salicylic acid.

DPPH scavenging rate (%) = [1 − (*A_x_* − *A_x_*_0_) /*A*_0_] × 100% (7)

*A_x_*: DPPH solution + sample solution;

*A_x_*_0_: sample solution + absolute ethanol;

*A*_0_: DPPH solution + distilled water.

### 2.10. Determination of Gastrointestinal Tolerance of Bovine-Derived LAB Fermented Milks

The gastrointestinal tolerance of bovine-derived LAB fermented milk was determined according to the method described by Wang et al. [17], with reference to the method of Vinyard for preparing simulated gastrointestinal digestive fluid (used within 24 h) with slight modifications. Briefly, 8 mL of deionized water was mixed with 1 mL of skim milk supernatant and stirred evenly. Simulated gastric digestive fluid: The pH of the mixture was adjusted to 2.0 with 1 mol/L HCl, and 0.01 g of pepsin was added. Deionized water was added to bring the total volume to 10 mL, followed by sterilization through a 0.22 μm microporous membrane filter. Simulated intestinal digestive fluid: The pH of the mixture was adjusted to 3.0 with 1 mol/L HCl, and 0.006 g of pepsin and 0.004 g of trypsin were added. Deionized water was added to bring the total volume to 10 mL, followed by sterilization through a 0.22 μm microporous membrane filter.

Simulated gastric fluid tolerance: Fermented milk (1%) was inoculated into the simulated gastric digestive fluid and subjected to simulated gastric digestion at 37 °C under anaerobic conditions. Samples were taken at 0, 2, and 4 h, and the absorbance at 600 nm was measured. Simulated intestinal fluid tolerance: Fermented milk (1%) was inoculated into the simulated intestinal digestive fluid and subjected to simulated intestinal digestion at 37 °C under anaerobic conditions. Samples were taken at 0, 2, 4, and 6 h, and the absorbance at 600 nm was measured.

### 2.11. In Vitro Colonic Fermentation and Microbial Community Structure of Different Bovine-Derived LAB Fermented Milks Using In Vitro Simmental Calf Feces

In vitro colonic fermentation was performed using a batch culture method to simulate the colonic conditions of Simmental calves. Fresh feces were collected and processed from Simmental calves to serve as the inoculum. The experiment was conducted under sterile and anaerobic conditions and divided into 4 groups: LpM, EhM, EfM, and CONM, with 3 replicates per group. Concentrations of short-chain fatty acids (SCFAs) were expressed as mmol/L. Blank control groups without bacterial inoculation were included in the in vitro fermentation system. The final SCFA concentrations were calculated after blank correction to eliminate background interference from the medium.

Simmental calves were fed 6 L of colostrum 1 day after birth and 4 L of whole milk per day from 2 to 7 days. On day 7, 4 healthy Simmental calves were selected, and 50 g of fresh fecal samples was collected from each calf by colonic sampling. Under anaerobic conditions, the fecal samples were mixed with sterile phosphate-buffered saline (PBS, pH 7.4) at a ratio of 1:6 (*w*/*v*) to obtain fecal slurry (Pfs). Fecal treatment liquid (Ftl) was prepared by mixing Pfs with reducing solution at a ratio of 1:5 (*v*/*v*). The reducing solution was prepared as follows: L-cysteine hydrochloride 2.52 g, 1 mol/L sodium hydroxide 16 mL, anhydrous sodium sulfide 2.56 g, and distilled water 380 mL. The medium composition of fermentation medium is shown in Table 2.

The in vitro colonic fermentation procedure was as follows: 3 mL of Ftl, 2 mL of bovine-derived LAB fermented milk, and 27 mL of in vitro fermentation medium were mixed. After thorough mixing, the mixture was incubated at 37 °C for 48 h. After fermentation, samples were centrifuged (3000 r/min, 30 min) and stored at −80 °C for later use. The fermentation supernatant was used to determine the SCFA content by ELISA kits.

The microbial composition of the fermentation precipitate was analyzed by 16S rRNA gene sequencing. Total genomic DNA was extracted from each sample using the E.Z.N.A. DNA Kit (Omega Bio-Tek, Norcross, GA, USA). The V3–V4 hypervariable region was amplified using barcoded forward primer 338F (5′-ACTCCTACGGGAGGCAGCAG-3′) and reverse primer 806R (5′-GGACTACHVGGGTWTCTAAT-3′). The amplicons were recovered by 2% agarose gel electrophoresis and purified using the AxyPrep DNA Gel Extraction Kit (Axygen Biosciences, Union City, CA, USA). Sequencing was performed by Shanghai Majorbio Bio-pharm Technology Co., Ltd. (Shanghai, China). Rarefaction curves, Venn diagrams of operational taxonomic units (OTUs), alpha and beta diversity analyses of the intestinal microbiota, changes in microbial species abundance, and correlation analyses between microbial communities and SCFA contents in the fermentation broth were conducted on the Majorbio Cloud Platform. For α-diversity, rarefaction curves were generated to assess sequencing coverage. The Good’s coverage index was calculated to evaluate sampling completeness. For β-diversity, a permutational dispersion test was conducted to check the homogeneity of group dispersions before PERMANOVA. LEfSe analysis was performed with an LDA threshold of 3.0 and a significance cutoff of *p* < 0.05.

### 2.12. Statistical Analysis

Following pre-processing in Microsoft Office, all data were analyzed by one-way analysis of variance (ANOVA) using SAS 9.4 (SAS Institute, Cary, NC, USA). Normality of residuals was assessed using the Shapiro–Wilk test, and homogeneity of variances was evaluated using Levene’s test in PROC GLM. When these assumptions were met, one-way analysis of variance (ANOVA) was conducted, followed by Tukey’s honestly significant difference (HSD) test for pairwise comparisons. For variables that violated the assumption of equal variances, Welch’s ANOVA was applied, and pairwise comparisons were conducted using the Games–Howell test, or the Tukey–Kramer test where appropriate. All tests were two-sided, and significance was set at *p* < 0.05, and extreme significance at *p* < 0.01.

## 3. Results

### 3.1. Growth and Acidification Curves of Strains in Different Bovine-Derived LAB Fermented Milks

The growth curves of the 3 LAB strains revealed their growth dynamics during raw milk fermentation, as shown in Figure 2A. The 3 strains Lp, Ef, and Eh exhibited similar growth trends: rapid growth before 12 h, slowed growth after 12 h, another rapid growth phase after 15 h, and stabilization after 21 h. The fermentation biomass of Lp and Eh strains was higher than that of Ef. The acid-producing capacity of the 3 LAB strains during raw milk fermentation is shown in Figure 2B. The results indicated that the Lp strain had a stronger acid-producing ability within the first 12 h, which gradually slowed down thereafter, with the final pH stabilizing at approximately 5. In contrast, Ef and Eh strains exhibited weaker acid-producing capacity within 12 h; however, after 12 h, their metabolism accelerated and acid production increased.

### 3.2. Nutrient Utilization Capacity of Different Bovine-Derived LAB

The nutrient utilization capacities of the different bovine-derived LAB strains are presented in Figure 2C. Compared with the Ef strain, both the Lp and Eh strains exhibited significant improvements in GOS and LOS utilization (*p* < 0.01), and the Lp strain was significantly higher than the Eh strain (*p* < 0.01). No significant differences were observed in the proteolytic properties among the 3 LAB strains (*p* = 0.192).

### 3.3. Probiotic Properties of LAB in Different Bovine-Derived LAB Fermented Milks

The probiotic properties of LAB in different bovine-derived LAB fermented milks are presented in Figure 2D–G. The water holding capacity and surface hydrophobicity rate of Lp in LpM were both significantly higher than those in EhM and EfM (*p* < 0.01). The auto-aggregation rate of Lp in LpM was significantly higher than that in EhM and EfM (*p* < 0.01). With respect to co-aggregation with pathogens, the co-aggregation rates of LAB in EhM and EfM with *Staphylococcus aureus*, *Escherichia coli*, and *Salmonella* were significantly higher than those in LpM; moreover, the co-aggregation rates of Eh in EhM with *S. aureus* and *Salmonella* were significantly higher than those in EfM (*p* < 0.01).

### 3.4. Antibacterial Activity and Antibiotic Sensitivity of Bovine-Derived LAB

The antibacterial activity of different bovine-derived LAB fermented milks is shown in Figure 2H. The LAB in all 3 fermented milks exhibited inhibitory effects against *Staphylococcus aureus*, *Escherichia coli*, and *Salmonella*, although the inhibitory capacities varied. Compared with LpM, the LAB in EfM and EhM showed significantly enhanced inhibitory effects against *Staphylococcus aureus*, *Escherichia coli*, and *Salmonella*; furthermore, Ef in EfM exhibited significantly higher inhibitory activity against these 3 pathogens than Eh in EhM.

The antibiotic sensitivity of different bovine-derived LAB fermented milks is presented in Table 3. For kanamycin, streptomycin, tetracycline, and doxycycline, the LAB in LpM, EfM, and EhM all showed high sensitivity. Lp in LpM showed high sensitivity to gentamicin, erythromycin, and ofloxacin, and intermediate sensitivity to ampicillin and lincomycin. Eh in EhM showed intermediate sensitivity to gentamicin, erythromycin, and lincomycin, and was resistant to ampicillin and ofloxacin. Ef in EfM showed intermediate sensitivity to erythromycin, and was resistant to gentamicin, ampicillin, ofloxacin, and lincomycin.

### 3.5. Active Substances in Fermented Milks Produced by Different Bovine-Derived LAB Strains

The RCRs of active substances in different bovine-derived LAB fermented milks before and after fermentation are presented in Figure 3A. The IgG retention rates of LpM and EhM were significantly higher than those of EfM (*p* < 0.01). Compared with LpM, the lactose retention rates of EfM and EhM were significantly increased (*p* < 0.01); moreover, the lactose retention rate of EfM was significantly higher than that of EhM (*p* < 0.01). With respect to SCFAs, the production rates of lactic acid and acetic acid in EhM were significantly higher than those in EfM (*p* < 0.01). The production rate of acetic acid in LpM was significantly higher than that in EfM (*p* < 0.01). The production rates of propionic acid and butyric acid in LpM were significantly higher than those in EhM and EfM (*p* = 0.041, *p* = 0.046).

### 3.6. Antioxidant Activity of Fermented Milks from Different Bovine-Derived LAB Strains

The antioxidant activity of active substances in different bovine-derived LAB fermented milks is shown in Figure 3B. EhM, LpM, Eh, and Lp exhibited a stepwise rise in DPPH radical scavenging activity, distinctly exceeding the values of EfM, Ef, and CONM. Both EfM and Ef had significantly greater DPPH scavenging rates than CONM (*p* < 0.01). Regarding hydroxyl radical scavenging, LpM, EhM, and EfM demonstrated marked enhancements over CONM, whereas Ef showed a significant decline (*p* < 0.01); moreover, the hydroxyl radical scavenging rate of LpM was significantly higher than those of EhM and EfM (*p* < 0.01).

### 3.7. Gastrointestinal Tolerance of Bovine-Derived LAB Fermented Milks

The survival rates of LAB in different bovine-derived LAB fermented milks after treatment with simulated gastrointestinal fluids are shown in Figure 3C,D. The survival rates of LAB in the three fermented milks exhibited different decreasing trends with prolonged treatment time. Among them, Lp in LpM showed the highest survival rate in simulated gastrointestinal fluids, while Eh in EhM exhibited an intermediate survival rate. Ef in EfM showed the fastest decline in survival rate.

### 3.8. In Vitro Fermentation Indices of Different Bovine-Derived LAB Fermented Milks in Simmental Calves

#### 3.8.1. Relative Change Rates of SCFAs in In Vitro Fermentation Broth of Different Bovine-Derived LAB Fermented Milks

The relative change rates of short-chain fatty acids in the in vitro fermentation broth of different bovine-derived LAB fermented milks are presented in Figure 3E. Compared with EfM, the production rates of lactic acid, acetic acid, propionic acid, and butyric acid in the in vitro fermentation broths of both LpM and EhM were significantly increased; moreover, the production rates of lactic acid, acetic acid, propionic acid, and butyric acid in the in vitro fermentation broth of EhM were significantly higher than those of EfM (*p* < 0.01).

#### 3.8.2. Effects of Different Bovine-Derived LAB Fermented Milks on the Composition and Diversity of Colonic Microbial Communities in Simmental Calves

##### Microbial Diversity Patterns

Sequencing analysis of the in vitro fermentation microbiota was performed to evaluate the effects of different bovine-derived LAB fermented milks on the composition and diversity of colonic microbial communities in Simmental calves. Venn analysis revealed that the LpM, EfM, and CONM groups contained three, one, and one unique amplicon sequence variants (ASVs), respectively, whereas the EhM group contained no unique ASVs (Figure 4A). All groups shared 10 ASVs, indicating the presence of a core microbiota during the in vitro fermentation process, while each LAB fermentation intervention resulted in a distinct microbial composition. Microbial alpha diversity analysis indicated that LAB fermented milks such as LpM and EfM significantly influenced the alpha diversity of fecal microbial communities during in vitro colonic fermentation inoculated with Simmental calf feces. Both the Shannon and Simpson indices showed that, compared with the CONM group, LpM and EfM significantly altered microbial richness or evenness, and significant differences were also observed between the LpM and EfM groups (*p* < 0.05) (Figure 4B,C). Principal coordinate analysis (PCoA) based on Bray–Curtis distances revealed significant differences in microbial community structure between the CONM and the LpM and EfM (R = 0.9028, *p* = 0.001). The CONM formed an independent cluster and was separated from the LpM and EfM groups along the principal coordinates, with these two axes accounting for 88.18% and 5.30% of the total variation, respectively. Although the CON showed some similarity with the EhM in the negative region of PC1, the two groups remained distinguishable (Figure 4D). This clear separation demonstrated that LAB fermented milks significantly altered the overall composition of the microbiota during in vitro fermentation.

##### Phylum- and Genus-Level Taxonomic Community Structure

At the phylum level, the fecal microbial communities in all groups were predominantly composed of *Pseudomonadota* and *Bacillota*. Compared with the other three groups, the EfM group exhibited a higher abundance of *Pseudomonadota* (79.16%) and a lower abundance of *Bacillota* (20.83%). The LpM group ranked second, while the EhM and CONM showed the most similar bacterial community compositions (Figure 5A). At the genus level, *Escherichia-Shigella* and *Enterococcus* were the absolutely dominant genera in the fecal microbial communities across all groups. Compared with the other groups, the EfM showed the highest abundance of *Escherichia-Shigella* (75.22%), which served as its characteristic flora. The EhM and CONM were highly similar in genus-level bacterial community composition (Figure 5B). Statistical analysis at the phylum level revealed significant changes, as shown in Figure 5C. The abundance of *Pseudomonadota* in the EfM was significantly higher than that in the other three groups (*p* < 0.05), while the abundance of *Bacillota* was significantly lower than that in the CONM (*p* < 0.05). As shown in Figure 5D, statistical analysis at the genus level also revealed significant changes. The abundances of *Escherichia-Shigella*, *Clostridium*, *Levilactobacillus*, and *Fusicatenibacter* in the EfM were significantly higher than those in the other three groups (*p* = 0.002), while the abundance of *Klebsiella* was significantly lower than those in the other three groups (*p* = 0.002). Compared with the CONM, the abundances of *Butyricicoccus* in the LpM, EfM, and EhM were significantly increased (*p* = 0.030). The abundance of *Enterococcus* in the LpM and EfM was significantly lower than that in the EhM and CONM (*p* < 0.01). The abundance of *Lactococcus* in the LpM and EhM was significantly higher than that in the EfM and CONM (*p* < 0.01). The abundance of *Methylobacterium* in the EfM and EhM was significantly higher than that in the LpM and CONM (*p* = 0.041).

##### Identification of Microbial Biomarkers via LEfSe Analysis

LEfSe analysis was performed to identify microbial taxa with significant abundance differences among groups, with the threshold for significant LDA score set at >3.0. The analysis revealed microbial biomarkers specific to each dietary regimen (Figure 6A). The LpM was mainly characterized by four unclassified taxa, including *g_Lactococcus*, *f_Streptococcaceae*, *f_Lachnospiraceae*, and *o_Lachnospirales*. In the EhM, *g_Enterococcus*, *f_Enterococcaceae*, *o_Lactobacillales*, *c_Bacilli*, *p_Bacillota*, and *g_Klebsiella* served as key biomarkers. The EfM exhibited a more complex biomarker profile, comprising *g_Escherichia-Shigella*, *c_Gammaproteobacteria*, *o_Enterobacterales*, *f_Enterobacteriaceae*, *p_Pseudomonadota*, *c_Clostridia*, *f_Clostridiaceae, o_Clostridiales*, and *g_Clostridium*. These findings suggest that specific bacterial taxa can act as potential biomarkers for colonic fermentation of LAB fermented milk.

##### MetaCyc Annotation Analysis

MetaCyc was used to annotate the abundance of metabolic pathways in the microbiota from in vitro fermentation fluids of different LAB-fermented milk (Figure 6B). The EhM and LpM showed high-abundance enrichment in carbon-fixation and membrane lipid synthesis pathways such as the Calvin cycle and phospholipid biosynthesis. The EfM was characterized by high expression of glycolysis and fatty-acid biosynthesis pathways, whereas the CONM maintained low abundance across all detected pathways.

##### Microbial Co-Expression Network Analysis

Figure 6C shows complex synergistic and competitive correlations among microorganisms across the three groups. The key nodes in the core network were *Fusicatenibacter*, *Lactococcus*, and *Escherichia-Shigella*. Among them, *Fusicatenibacter* exhibited significant positive correlations with *Lactococcus* and *Thomasclavelia*. By contrast, *Escherichia-Shigella* showed negative correlations with *Klebsiella* and *Methylobacterium*. The correlation patterns of core taxa among groups treated with different LAB-fermented milk in vitro indicated that different LAB-fermented milk reshaped the microbial community structure by regulating inter-species interactions.

##### Correlation Analysis Between Microbial Genera and Key Volatile Fatty Acids

Spearman correlation analysis was performed to clarify the relationships between microbial genera with differential abundances and key SCFAs (Figure 6D). Acetic acid and butyric acid concentrations showed extremely significant positive correlations with multiple genera, including *Butyricicoccus*, *Lactococcus*, and *Klebsiella*, suggesting that these genera are core contributors to SCFA biosynthesis. Propionic acid concentration was significantly positively correlated with the abundance of *Butyricicoccus*. Lactic acid concentration displayed an extremely significant positive correlation with *Butyricicoccus* abundance, as well as a significant positive correlation with the abundances of *Lactococcus* and *Klebsiella*. These results confirmed that structural differences in microbial communities across different treatment groups directly determined the biosynthesis levels of SCFAs.

## 4. Discussion

LOS is a disaccharide composed of glucose and GOS linked by a β-1,4-glycosidic bond, which serves as an energy source for the growth and reproduction of LAB during raw milk fermentation. The hydrolysis of lactose into glucose and GOS constitutes the initial step for fermentation and acid production by LAB. LAB can convert lactose into lactic acid via lactic-acid fermentation, which not only reduces the pH value but also drives structural changes in milk matrix. In the present study, the lactose-utilizing capacity of Lp and Eh strains was significantly higher than that of Ef, suggesting that Lp and Eh could hydrolyze lactose efficiently to initiate fermentation. β-galactosidase is a key enzyme responsible for LOS hydrolysis. Abdel et al. reported that β-galactosidase isolated from fermented milk of *Lacticaseibacillus paracasei* MK852178 markedly reduced the lactose content of yogurt [24]. Tarrah et al. revealed that the genome of *Lacticaseibacillus paracasei* DTA72 encoded 80 proteins related to carbohydrate-active enzymes, indicating its robust carbohydrate-metabolizing capacity [25]. Bovine milk oligosaccharides are dominated by sialylated oligosaccharides [26]. *Enterococcus hirae* F42 is sensitive to oligosaccharides, and synergistic effects exist between them. Oligosaccharides can promote the growth and metabolism of strains. Meanwhile, by increasing the abundance of key metabolites such as carboxylic acids during fermentation, oligosaccharides can up-regulate carbohydrate-utilization pathways including glycolysis and GOS metabolism, thereby altering the sensory properties of milk products [27]. Nevertheless, Kongo et al. found that cheese-derived *Enterococcus* strains exhibited high β-galactosidase activity [28]. Growth curves and acid-production curves are vital indicators for evaluating the viability, fermentation efficiency and quality of LAB-fermented milk. In this experiment, Lp, Eh and Ef exhibited similar growth dynamics during fermentation, with the biomass of Lp and Eh being higher than that of Ef. In terms of pH, Lp fermented milk displayed a faster acid-production rate compared with Ef and Eh fermented milk, implying that Lp possessed a prominent advantage in converting carbohydrates into lactic acid.

Water-holding capacity is a critical indicator for evaluating the textural properties of fermented milk. Fermentation products can fully interact with casein to promote casein aggregation, thereby improving the water-holding capacity of fermented milk. Probiotics produce exopolysaccharides during fermentation, facilitating the formation of porous structures and reducing whey separation. Accordingly, probiotic fermentation can enhance product quality by increasing water-holding capacity. In the present study, the water-coagulating capacity, surface hydrophobicity and auto-aggregation rate of strain Lp in the LpM were higher than those in the EhM and EfM. In line with previous studies, this superior water-holding performance may be associated with exopolysaccharides produced during fermentation [29]. This finding was consistent with Sharma et al., who reported that milk-originated *Lacticaseibacillus paracasei* CD4 exhibited high surface hydrophobicity and auto-aggregation ability [30]. Rocha-Mendoza et al. further demonstrated that *Lacticaseibacillus paracasei* OSU-PECh-3B up-regulated the expression of *Cnb* and *EF-Tu* in medium supplemented with milk phospholipids, enhancing its adhesion to Caco-2 cells [31]. This indicated that milk matrix could modulate adhesive properties by regulating protein-coding genes of strains. Saini et al. reported that *Enterococcus hirae* possessed certain surface hydrophobicity, possibly because it synthesized bacteriocin such as Enterocin BNT6 to alter surface-charge distribution and further modulate hydrophobicity [32]. In addition, *Enterococcus faecalis* displayed strong intercellular aggregation and water-retention capacity via exopolysaccharide-like phenotypes [33].

LAB generally exert stronger antagonistic effects against Gram-positive pathogens such as *Staphylococcus aureus* than against Gram-negative pathogens including *Escherichia coli* and *Salmonella* spp. [34]. Bovine-origin lactic-acid bacteria exert antibacterial effects by lowering environmental pH via organic-acid production (e.g., lactic acid), secreting bacteriocins and other antimicrobial peptides, conducting competitive exclusion and interfering with pathogen biofilm formation [35]. Co-aggregation between LAB and pathogens reflects specific cell-to-cell binding capacity, which is partially correlated with pathogen-inhibitory activity. In this study, LAB in EhM and EfM showed higher pathogen-inhibitory activity and co-aggregation rates toward pathogens than those in the LpM, which was in accordance with Elnar et al., where *Enterococcus faecalis* CAUM157 exhibited strong inhibition against *Listeria* [36]. Previous research revealed that chicken-derived *Enterococcus faecalis* LX10 up-regulated adhesion-related genes (*znuA*, *lepB*, *hssA*, *adhE*, *EbpA*, *Lap*) and regulatory gene *BfmRS*, providing molecular bridges to cell-wall components of harmful bacteria such as *Enterobacter* and promoting co-aggregation with pathogens [37]. Moreover, *Enterococcus faecalis* could trigger antibacterial activity against *E. coli* through direct cell–cell contact [38]. Nevertheless, contradictory results have been documented. Zhong et al. found that *Lacticaseibacillus paracasei* ProSci101 enriched glycerophospholipid metabolites and significantly suppressed the growth of pathogenic bacteria including *Staphylococcus aureus* and *E. coli* [39]. The reasons for the relatively low co-aggregation rate of LpM warrant further investigation.

Antibiotic-susceptibility profiling is essential for safety management of LAB species. In this experiment, strain Lp from LpM was highly susceptible to kanamycin, streptomycin, tetracycline and doxycycline, consistent with Comunian et al., in which 94 out of 121 *Lacticaseibacillus paracasei* isolates were sensitive to tetracycline [40]. Lp showed intermediate susceptibility to ampicillin and lincomycin. Shahverdi et al. observed that some *Lacticaseibacillus paracasei* strains were susceptible to streptomycin but exhibited moderate resistance to kanamycin [41]. As stated by Kim et al., aminoglycoside resistance in *Lacticaseibacillus paracasei* can be attributed to cell-wall impermeability, efflux-pump systems, drug transporters and antibiotic-resistance genes [42]. Strain Eh in the EhM displayed intermediate susceptibility to gentamicin, erythromycin and lincomycin, whereas it was resistant to ampicillin and ofloxacin. Bovine-derived *Enterococcus hirae* isolates are usually susceptible to high-concentration gentamicin [43]. Xi et al. reported that *Enterococcus hirae* ZJ02 exhibited resistance to lincomycin and susceptibility to chloramphenicol and ampicillin [13]. Strain Ef in the EfM showed intermediate susceptibility to erythromycin and resistance to ampicillin, which agreed with Channaiah et al. for insect-origin *Enterococcus faecalis* [44]. On the contrary, Dec et al. found wild-animal-sourced *Enterococcus faecalis* was resistant to tetracycline, ofloxacin and erythromycin but susceptible to ampicillin and streptomycin; such discrepancies are also ascribed to strain-source variations [45].

Fermented-milk-derived IgG can prevent colonic lipopolysaccharide leakage in calves by regulating intestinal microbiota and stimulating SCFA production, thereby inhibiting pathogen adhesion. Meanwhile, IgG can trigger immune responses by promoting the uptake of IgG-pathogen immune complexes via Fc receptors. Metabolites including residual LOS, GOS and glucose generated from LOS fermentation by LAB can positively modulate the intestinal microbiota of calves, increase the relative abundance of beneficial taxa such as *Lacticaseibacillus*, *Akkermansia* and *Faecalibacterium*, and reduce the relative abundance of harmful genera such as *Clostridium* [46]. Such modulatory effects are accompanied by elevated levels of acetate, propionate and lactate [47]. Vitellio et al. reported that LOS produced by LAB could selectively stimulate commensal bacteria in healthy intestines and suppress colonic pathogens [48]. SCFAs impair membrane integrity and metabolic function of intestinal pathogenic bacteria including *Enterococcus* and *Escherichia coli* in calves [49]. In the present study, higher SCFA-production efficiency and IgG retention rate were observed in the LpM compared with the EfM. The mechanisms underlying these differences in IgG retention remain unclear. Both lactate- and acetate-production rates as well as IgG retention rates were higher in the EhM than in the EfM. This result is comparable to previous findings that strain *QU 50* efficiently produces L-lactic acid via homofermentation using xylose. Compared with Ef, Lp shows higher antibiotic susceptibility and possesses more specialized metabolic pathways, which helps stabilize the fermentative environment and preserve IgG. Nevertheless, inconsistent observations have been reported: rumen-derived *Enterococcus faecalis* S.156 from cattle can produce large amounts of L-lactic acid with a conversion rate up to 90% [50]. In this work, EfM and EhM exhibited higher lactose-retention rates than LpM. This pattern agrees with Sakoui et al., who found that *Enterococcus faecium* possesses moderate β-galactosidase activity yet shows a moderate lactic-acid-producing rate [51]. Zhong et al. identified a complete lactose-metabolism gene set including lacF, lacA/B, lacD, lacG and lacC in milk-origin *Enterococcus faecalis* [52]. The conservation of this pathway supports its adaptive strategy for the milk-associated niche.

ROS are by-products of normal cellular metabolism in animals. Excessive ROS trigger lipid peroxidation, protein structural damage, mitochondrial dysfunction and eventually cell apoptosis. Due to their hydrophobic properties, whey-protein hydrolysates from bovine milk exert antioxidant activity by efficiently scavenging ROS and free radicals. Casein hydrolysates from bovine milk also prevent lipid peroxidation, because small peptides derived from casein exhibit strong scavenging capacity against DPPH, hydrogen-peroxide radicals and superoxide anions. All three LAB-fermented milk samples in this study displayed antioxidant activity. During fermentation, *Lacticaseibacillus paracasei* generates multiple antioxidant substances, such as hydrogen peroxide and superoxide dismutase, and elevates the antioxidant capacity of milk via polyphenol-related metabolites. Shi et al. demonstrated that cheese-derived *Lacticaseibacillus paracasei* strains possess DPPH-scavenging capacity comparable to that of *Lacticaseibacillus rhamnosus* GG [53]. Probiotic fermented milk must withstand harsh digestive-tract conditions including strong gastric acid, pepsin and pancreatin before reaching the calf intestine and exerting beneficial effects. The three fermented-milk groups in our experiment exhibited distinct degrees of gastrointestinal tolerance. These results are consistent with Cao et al., who reported high gastric- and intestinal-fluid tolerance in food-origin *Enterococcus hirae* YC07 [54]. Similarly, Rwubuzizi et al. found that fermented-vegetable-derived *Enterococcus faecalis* ST11BG shows potent antioxidant activity and high stability under simulated gastric and duodenal conditions [55].

The intestinal microbiota of calves represents a promising target for diarrhea management and treatment. Calf intestinal microbiota is predominantly composed of anaerobic bacteria. The Yeast extract, Casitone, and Fatty Acids medium (YCFA) anaerobic medium provides favorable conditions for microbiota homeostasis by supplying growth factors, antioxidants, volatile fatty acids and vitamins [56]. SCFAs serve as primary energy sources for intestinal epithelial cells [57]. Specifically, acetate acts as a substrate for hepatic cholesterol and fatty-acid synthesis and modulates ileal contraction; propionate inhibits hepatic lipogenesis and exerts anti-inflammatory and anti-carcinogenic effects; and butyrate is preferentially utilized as energy by intestinal mucosa and enhances intestinal-barrier function. Alpha-diversity indices reflect the richness and diversity of intestinal microbial communities. The Shannon and Simpson indices are commonly applied markers for microbial diversity: higher Shannon index values and lower Simpson index values indicate higher microbial diversity. Figure 7 summarizes the regulatory mechanisms of three bovine-derived LAB fermented milks in an in vitro colonic fermentation model. In the present trial, LpM and EfM showed significantly higher α-diversity, Shannon index and Simpson index than other groups, suggesting that fermented milk prepared with Lp and Ef can improve gut microbial richness in calves. *Butyricicoccus pullicaecorum* can up-regulate tight-junction proteins via butyrate production, counteracting TNBS-induced reduction in transepithelial electrical resistance and excessive IL-8 secretion in the intestine [58]. The order *Lactobacillales* can reduce diarrhea incidence by enriching intestinal yeasts and lactic-acid bacteria and elevating propionate concentrations [59]. Jaramillo et al. demonstrated that cell-free supernatant from *Lactococcus* cultures inhibits adhesion of *Escherichia coli* 21259 and down-regulates expression of the virulence gene *faeG* in the gut of young animals [60]. In our study, the relative abundances of *Butyricicoccus pullicaecorum* and *Lactococcus* were higher in LpM, EhM and EfM than in the CONM, implying that the three fermented-milk products possess probiotic potential via stimulating intestinal SCFA synthesis. Beckman et al. reported that *Klebsiella* and *Enterococcus* exhibit strong biofilm-forming capacity [61]. Biofilms are complex structures built from extracellular polymeric substances, which generate hypoxic or micro-aerobic microenvironments suitable for anaerobic fermentation—a major pathway for acetate and butyrate biosynthesis. The significantly decreased abundances of *Klebsiella* and *Enterococcus* in the EfM indicated its weaker capacity for acetate and butyrate production relative to Lp and Eh, which matches our phenotypic observations. *Streptococcaceae* and *Lachnospiraceae* are typical probiotic taxa in fermented milk and showed pronounced proliferation especially following Lp fermentation. Members of *Streptococcaceae* boost intestinal SCFA generation and increase intestinal IgA levels [62]. *Lachnospiraceae* can alleviate pathogen-caused infection by enhancing antibiotic efflux and reducing bacterial antibiotic susceptibility [63]. In our assay, *Lachnospiraceae* dominated in intestinal fermentation samples of the LpM, in line with Mao et al., where Lp increased colonic relative abundances of *Ruminococcaceae* UCG-014 and *Lachnospiraceae* UCG_006 [64]. Collectively, Lp remodels intestinal microbiota and restrains pathogen colonization by enriching beneficial taxa such as *Lachnospiraceae*. These strain-specific differences in microbiota-modulating capacity may be associated with their distinct abilities to utilize milk-derived substrates such as LOS and GOS; however, this interpretation requires further validation. Diarrheic intestinal microecology is characterized by high abundances of *Escherichia-Shigella* and *Klebsiella-Enterococcus* [65]. *Enterococcus* is classified as an opportunistic pathogen because it may harbor virulence factors including haemolysin, adhesins and antibiotic-resistance genes. Fan et al. reported that *Bacillus* not only elevates the abundances of *Enterococcus* and *Lacticaseibacillus* while suppressing *Campylobacter* and *Escherichia-Shigella* but also alleviates diarrhea in goats by lowering pro-inflammatory cytokines and increasing anti-inflammatory mediators [66]. Although EhM and EfM increased *Enterococcus* abundance in this experiment, they still inhibited the proliferation of diarrhea-associated pathogenic bacteria. The LpM had the lowest abundance of *Escherichia-Shigella* among the three treatment groups, confirming the superior performance of Lp against pathogenic colonization. This inhibitory effect against pathogenic colonization is consistent with the in vitro antibacterial activity of Lp observed in this study. All three fermented-milk formulations reduced the abundance of pathogenic bacteria to varying extents, with Lp showing the strongest inhibitory effect, consistent with the results of in vitro antibacterial assays. Partial enrichment of diarrhea-related bacteria in EhM and EfM reminds us that potential intestinal risks of Eh and Ef cannot be neglected despite their antibacterial activity.

## 5. Limitations

Several limitations of this study should be acknowledged. First, all experiments were performed in vitro, including the simulated colonic fermentation model; therefore, the probiotic potential of Lp and the implications regarding calf diarrhea inferred from this study remain preliminary and require confirmation in in vivo trials in calves. In particular, the pathogenic risks associated with Eh and Ef (e.g., enrichment of *Enterococcus* observed in the fermentation model) should be carefully evaluated in animal models before any further application. Second, the fecal fermentation model, although widely used for screening, cannot fully reproduce the complex physiological environment of the calf gastrointestinal tract, including host immune responses, epithelial interactions and dynamic gut physiology; accordingly, the microbiota-modulating effects observed here should be interpreted as indicative rather than conclusive. Third, the strain-specific differences in lactose-utilizing efficiency and the proposed relationships between substrate utilization and microbiota modulation were inferred from phenotypic and 16S rRNA sequencing data alone. Multi-omics approaches are needed to dissect the metabolic regulatory mechanisms underlying the distinct functional phenotypes of the three strains. Future studies combining in vivo challenge trials in calves with multi-omics analyses will be essential to verify these hypotheses and provide a reliable basis for strain screening in the development of specialty fermented milk targeting calf diarrhea.

## 6. Conclusions

In conclusion, three bovine-derived LAB strains (Lp, Eh, and Ef) isolated from feces of healthy Simmental calves exhibited distinct functional performances when fermented in pasteurized milk. Strain Lp represents a promising candidate strain for preparing bovine-origin LAB fermented milk with the potential to prevent calf diarrhea based on in vitro screening results. Under milk fermentation, Lp displayed superior GOS and LOS utilization. LpM had better water-holding capacity, cell-surface hydrophobicity and auto-aggregation ability, as well as the strongest DPPH and hydroxyl-radical-scavenging antioxidant activity. By contrast, Eh and Ef showed stronger co-aggregation capacity against pathogenic bacteria including *Staphylococcus aureus*, *Escherichia coli*, and *Salmonella*, with Ef demonstrating the highest antibacterial potency. All three strains were highly susceptible to kanamycin, streptomycin, tetracycline and doxycycline; nevertheless, Eh and Ef exhibited intermediate sensitivity or resistance to several other antibiotics. In the in vitro colonic fermentation system, LpM promoted the enrichment of beneficial taxa such as *Butyricicoccus pullicaecorum*, *Lactococcus*, *Streptococcaceae* and *Lachnospiraceae* and yielded higher SCFA concentrations. Although fermented milks prepared with Eh and Ef could enrich potential beneficial *Bacillus*, they also increased the relative abundance of opportunistic pathogens including *Escherichia-Shigella*, *Klebsiella*, and *Enterococcus*. Further evaluation is required before Eh and Ef can be adopted as candidate strains for fermented milk against calf diarrhea. However, these findings are limited to in vitro assays; further in vivo feeding trials are required to verify their practical effects on diarrhea prevention in calves.

## Figures and Tables

**Figure 1:**
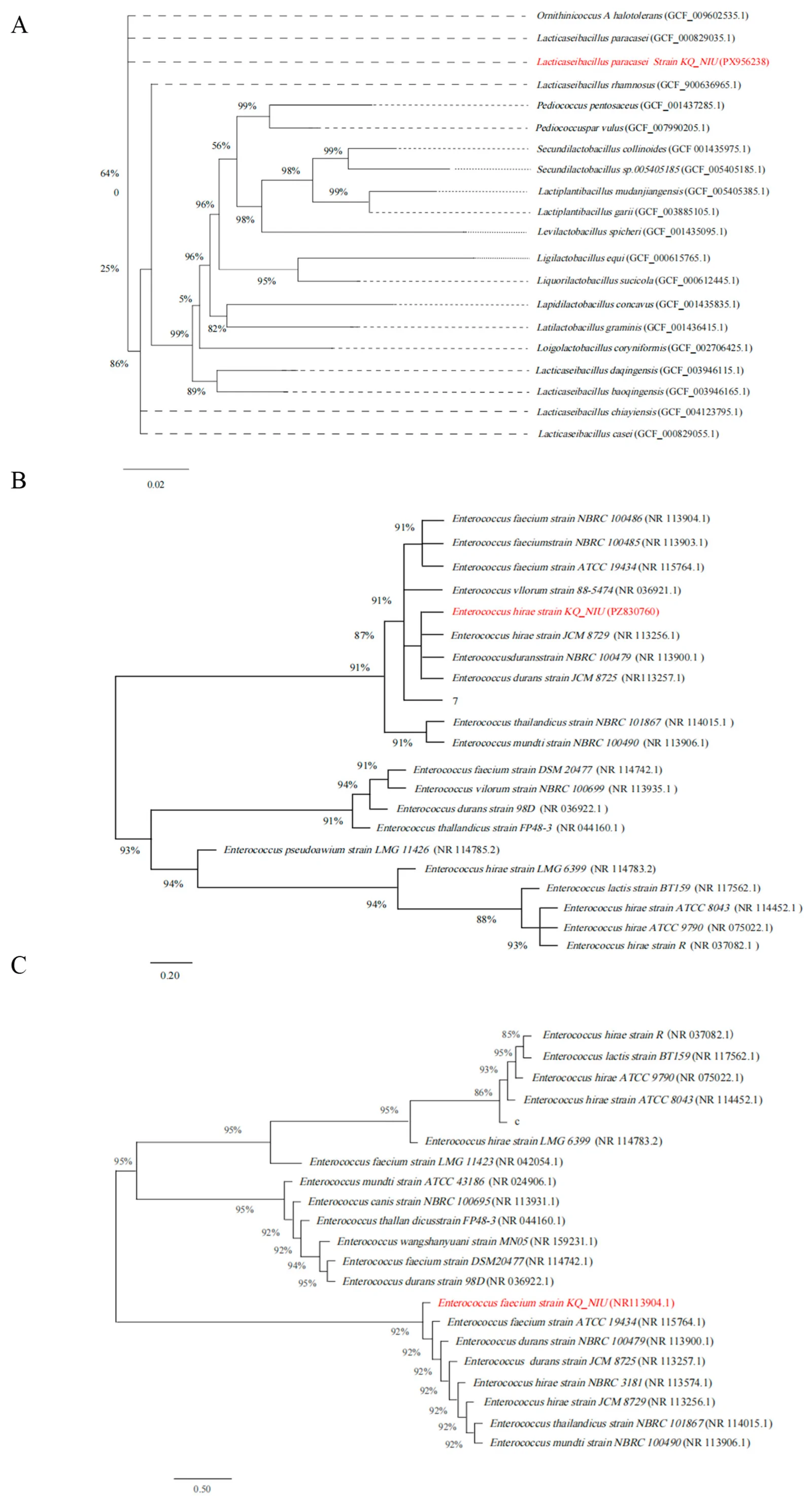
Phylogenetic tree constructed based on the 16S rRNA gene sequences of lactic acid bacteria from calf intestines. The numbers in parentheses represent the GenBank accession numbers of the strains. The numbers at the branch nodes indicate bootstrap values, representing the percentage of confidence for the phylogenetic tree branches. The scale bar of 0.01 indicates 1% sequence divergence. (**A**) phylogenetic tree of Lp [6]; (**B**) phylogenetic tree of Eh; (**C**) phylogenetic tree of Ef.

**Figure 2:**
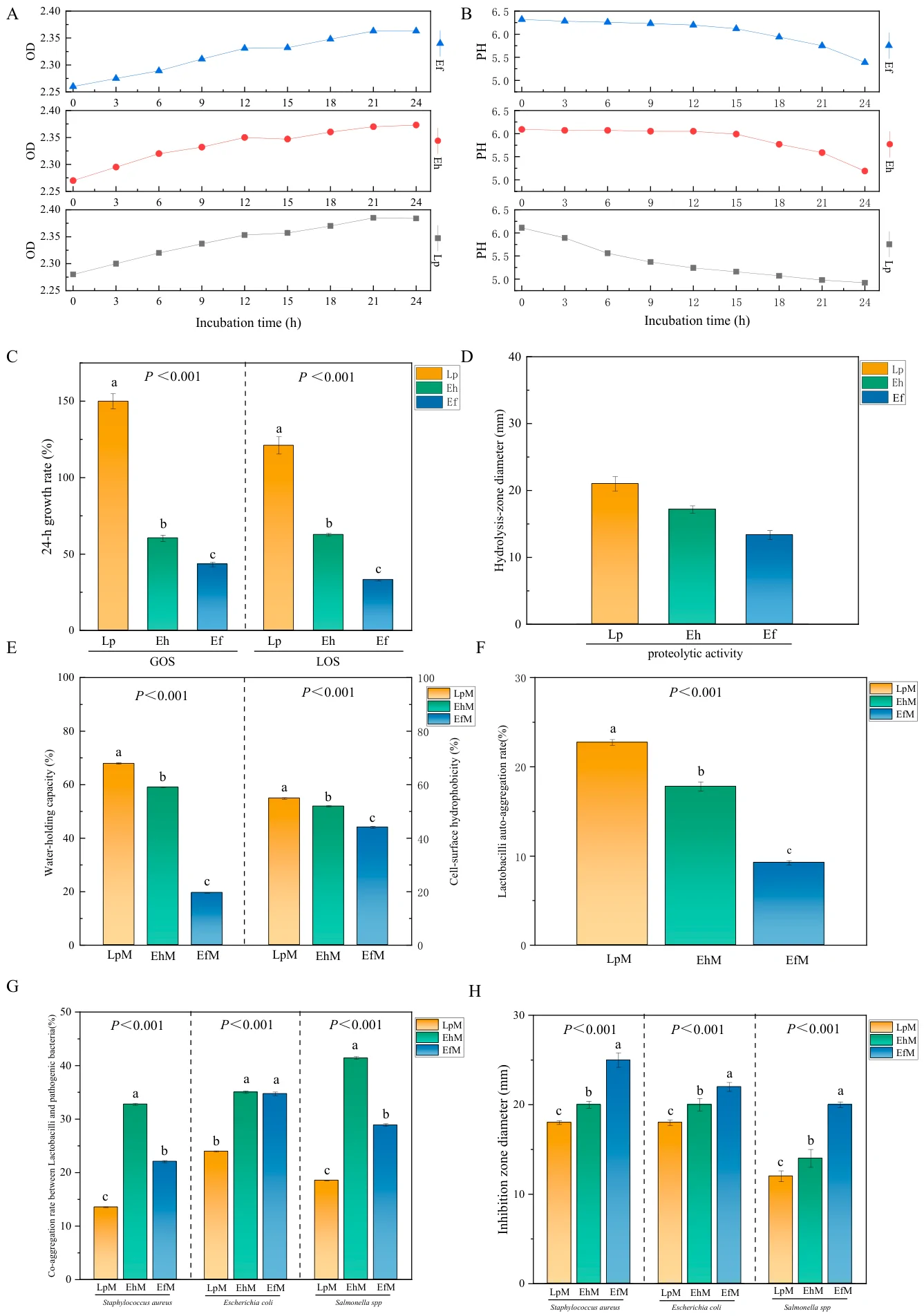
Growth dynamics, nutrient utilization, probiotic properties, and antibacterial activity of bovine-derived LAB and fermented milks. (**A**) Growth curves of Lp, Eh, and Ef during raw milk fermentation. (**B**) Acidification curves of Lp, Eh, and Ef during raw milk fermentation. Growth was monitored in raw milk, not MRS medium. The monotonous growth pattern is an inherent feature of raw milk fermentation. (**C**) Utilization capacity of Lp, Eh, and Ef for GOS and LOS. (**D**) Proteolytic activity of Lp, Eh, and Ef determined. (**E**) Water holding capacity and surface hydrophobicity of LAB in LpM, EhM, and EfM. (**F**) Auto-aggregation rate of LAB in LpM, EhM, and EfM. (**G**) Co-aggregation rate of LAB in LpM, EhM, and EfM with *Staphylococcus aureus*, *Escherichia coli*, and *Salmonella*. (**H**) Antibacterial activity of LpM, EhM, and EfM against *Staphylococcus aureus*, *Escherichia coli*, and *Salmonella*. Different lowercase letters (a, b, c) above the bars indicate significant (*p* < 0.05) or extremely significant differences (*p* < 0.01) among groups.

**Figure 3:**
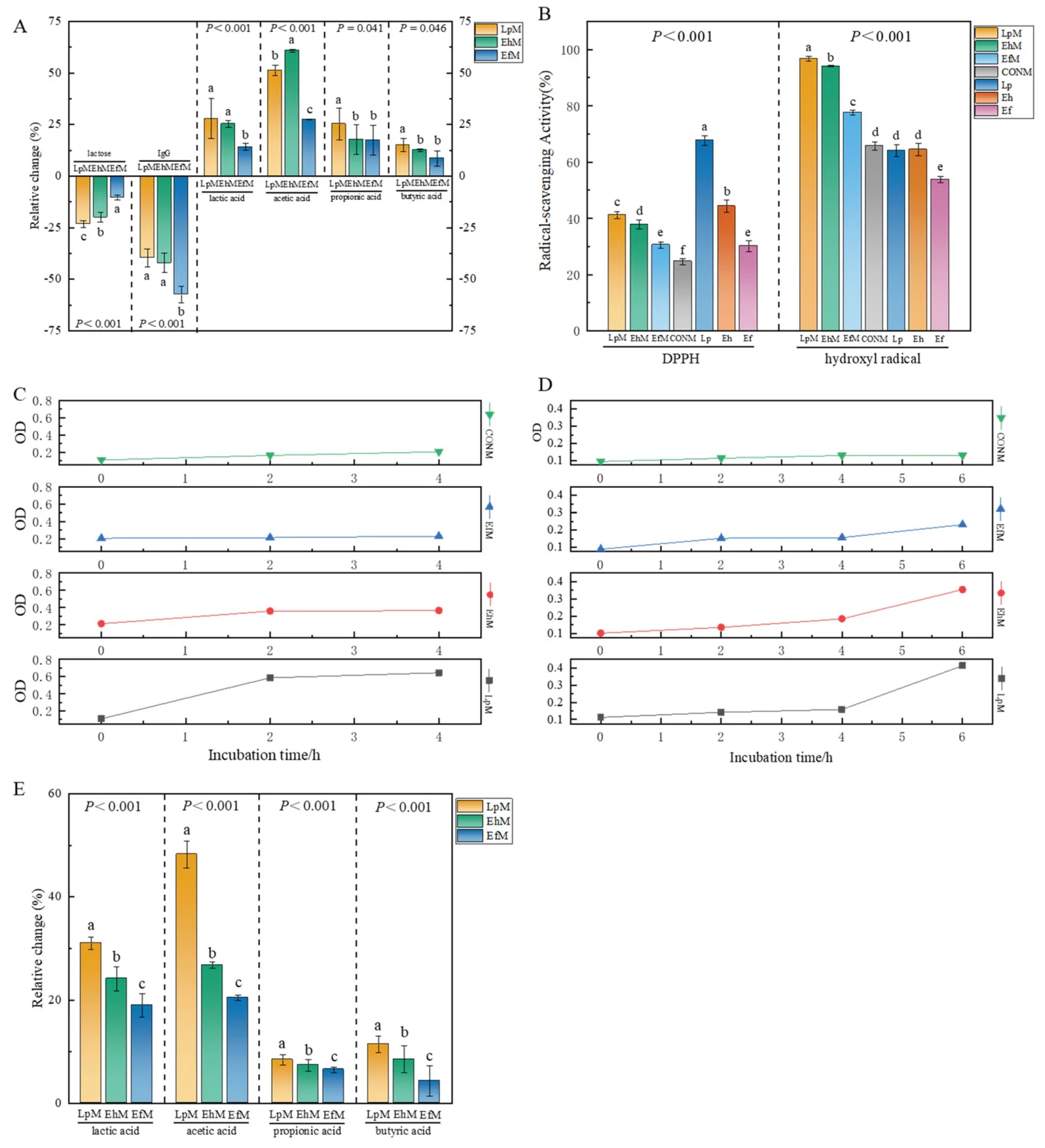
Active substances, antioxidant activity, gastrointestinal tolerance, and SCFAs production of different bovine-derived LAB fermented milks. (**A**) RCR of active substances in LpM, EhM, and EfM. (**B**) Hydroxyl radical and DPPH radical scavenging activities of CONM, Lp, Eh, Ef, LpM, EhM, and EfM. (**C**) *OD*_600_ changes in CONM, LpM, EhM, and EfM in simulated gastric fluid. (**D**) *OD*_600_ changes in CONM, LpM, EhM, and EfM in simulated intestinal fluid. (**E**) Relative change rate of SCFA in in vitro colonic fermentation broth. Different lowercase letters (a, b, c, d, e) above the bars indicate significant (*p* < 0.05) or extremely significant differences (*p* < 0.01) among groups.

**Figure 4:**
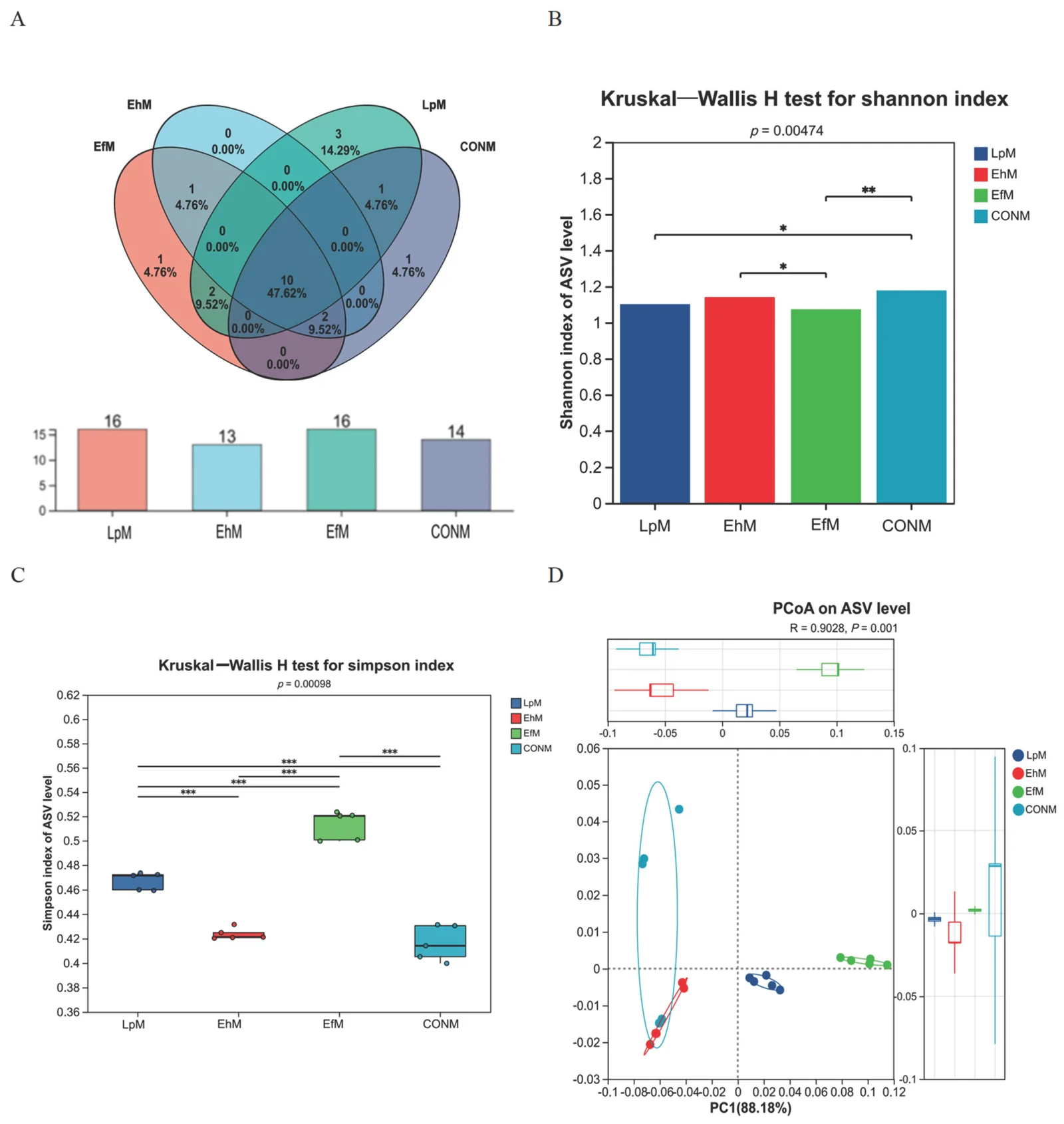
Microbial diversity patterns of in vitro colonic fermentation microbiota in response to different bovine-derived LAB fermented milks. (**A**) Venn diagram showing unique and shared ASVs among LpM, EhM, EfM, and CONM. (**B**) Shannon index of ASV level across the 4 groups. (**C**) Simpson index of ASV level across the 4 groups. (**D**) Principal coordinate analysis (PCoA) based on Bray–Curtis distances at the ASV level. Asterisks indicate significant differences, where * means *p* < 0.05, ** means *p* < 0.01, and *** means *p* < 0.001.

**Figure 5:**
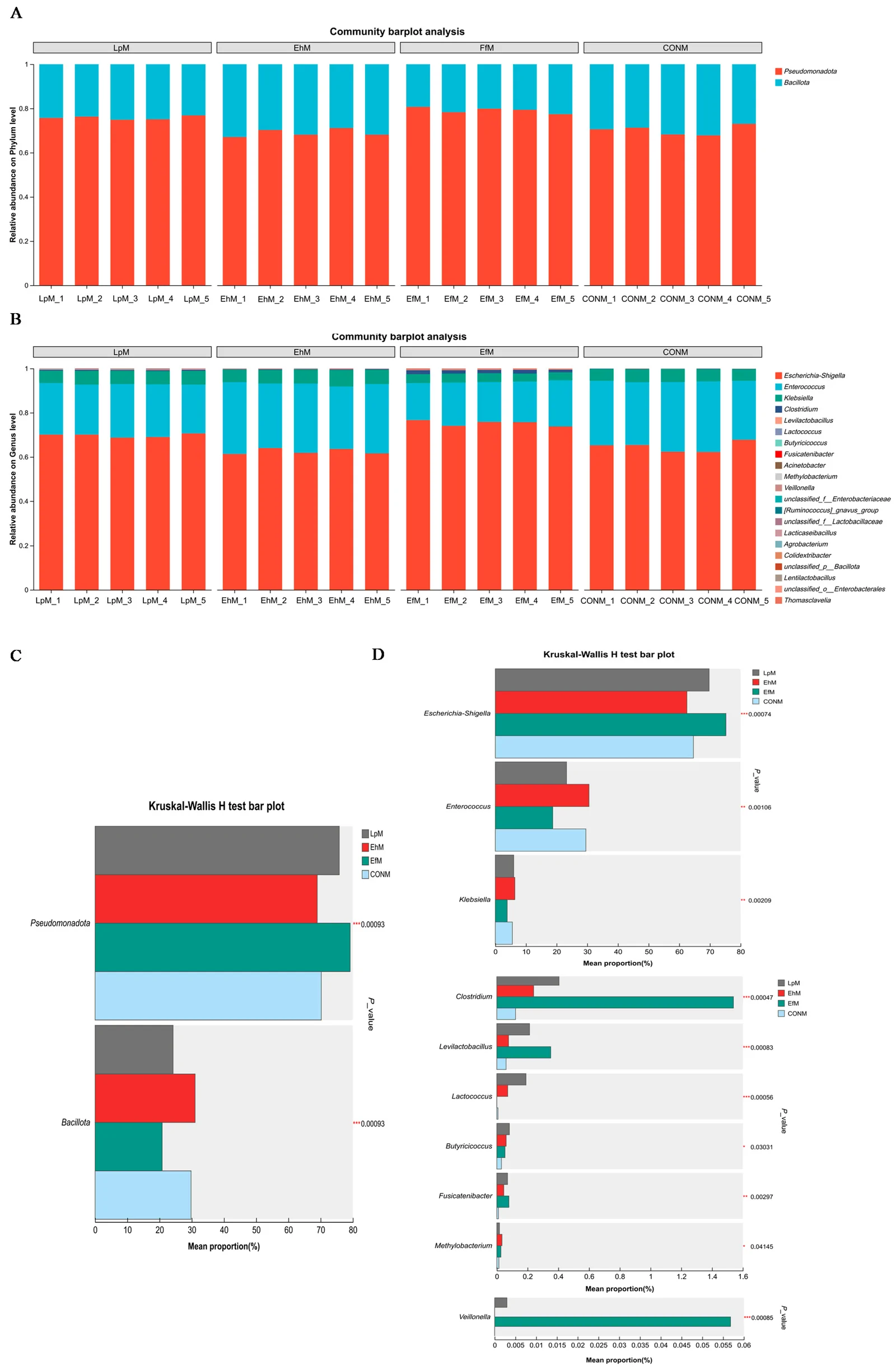
Microbiome composition and differential taxa analysis. (**A**) Relative abundance of microbial communities at the phylum level. (**B**) Relative abundance of microbial communities at the genus level. (**C**) Kruskal–Wallis H-test bar plot for differential taxa at the phylum level. (**D**) Kruskal–Wallis H-test bar plot for differential taxa at the genus level. Asterisks indicate significant differences, where * means *p* < 0.05, ** means *p* < 0.01, and *** means *p* < 0.001.

**Figure 6:**
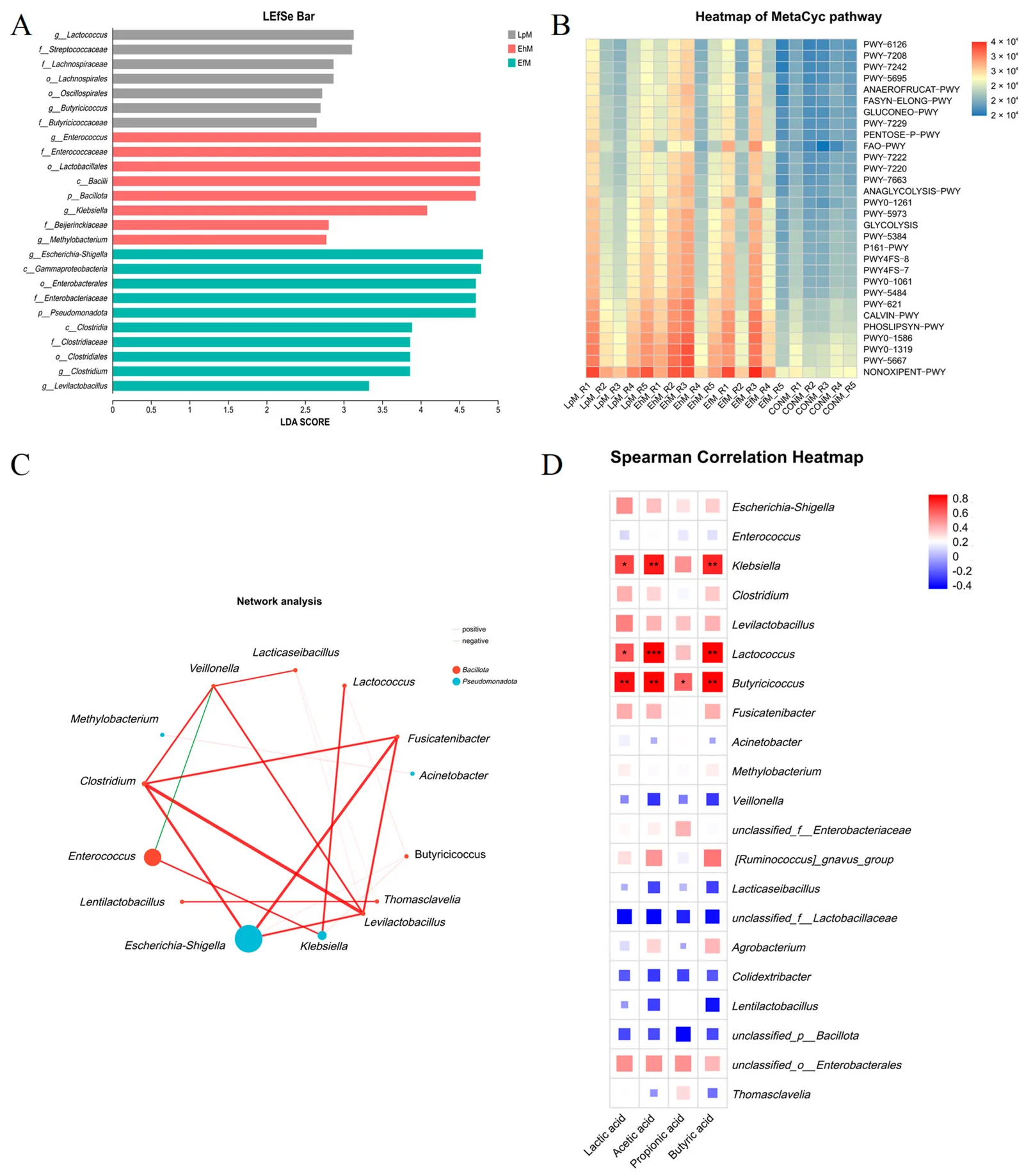
Analysis of microbial biomarkers, metabolic pathways, inter-taxa interactions and correlations between microbiota and SCFAs. (**A**) LEfSe bar plot showing microbial biomarkers with LDA score > 3.0 among different groups. Gray: LpM; red: EhM; green: EfM. (**B**) Heatmap of MetaCyc metabolic pathway abundance in in vitro fermentation samples. (**C**) Microbial co-occurrence network analysis. Red lines represent positive correlations; green lines represent negative correlations. Node size corresponds to the relative abundance of taxa. Nodes are colored according to phylum level: red, *Bacillota*; blue, *Pseudomonadota*. (**D**) Spearman correlation heatmap between microbial genera and SCFAs. Asterisks indicate significant differences, where * means *p* < 0.05, ** means *p* < 0.01, and *** means *p* < 0.001.

**Figure 7:**
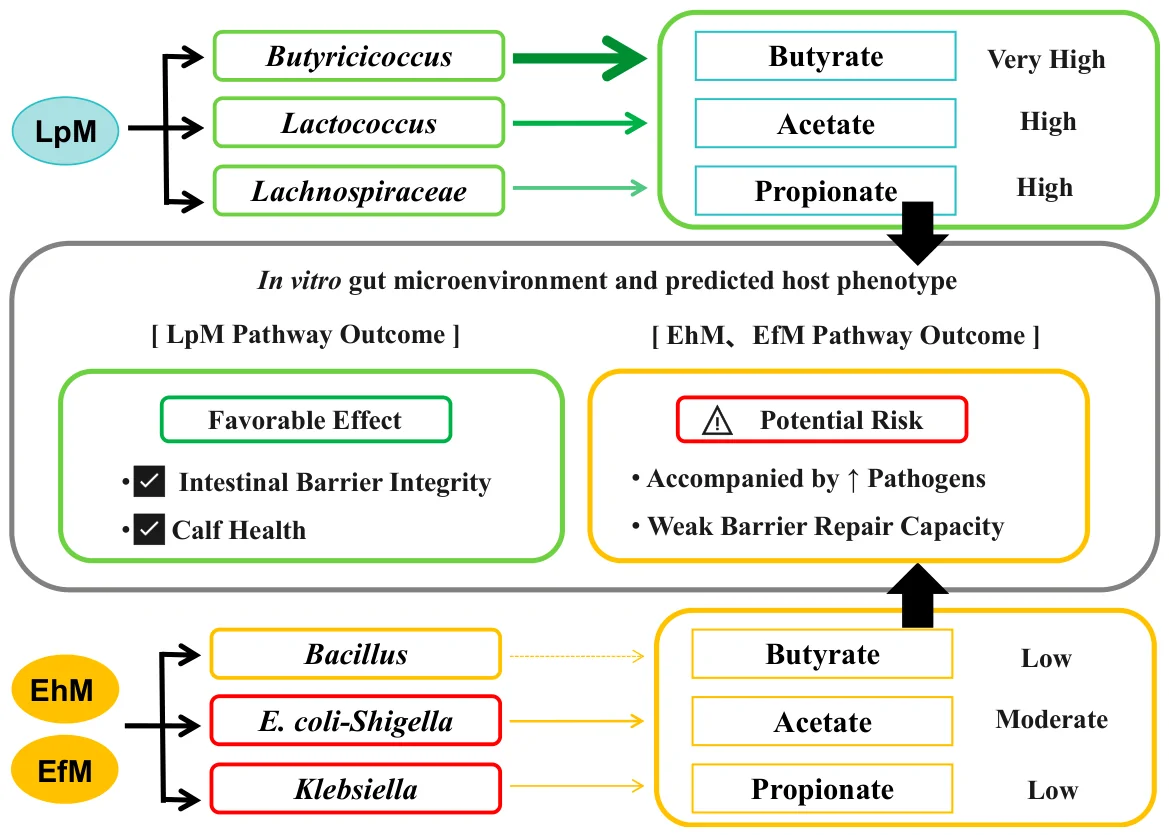
Schematic diagram illustrating the regulatory mechanism of 3 bovine-derived LAB fermented milks (LpM, EhM, EfM) in an in vitro colonic fermentation model. Green boxes denote beneficial microbial taxa and high SCFA levels; yellow boxes denote moderate microbial responses; red boxes denote opportunistic pathogens. The green rounded box represents beneficial effect, and the red rounded box represents potential risk. Thick solid arrows represent strong regulatory effects, and thin dashed arrows represent weak or limited effects.

**Table 1:** Criteria for antibiotic susceptibility determination [6].

Antibiotic Name	Drug Content in Paper (μg/Paper)	Diameter of the Inhibition Zone (mm)			Sensitivity Classification ^(1)^
		Sensitive	Indicates	Resistant	
Kanamycin	30	≥18	15~17	≤14	I
Streptomycin	10	≥15	12~14	≤11	I
Gentamicin	10	≥15	13~14	≤12	S
Tetracyciine	30	≥19	15~18	≤14	I
Doxycycline	30	≥19	15~18	≤14	S
Erythromycin	15	≥23	14~22	≤13	I
Ampicillin	10	≥17	13~16	≤13	I
Ofloxacin	5	≥21	16~20	≤15	I
Lincomycin	20	≥21	15~20	≤14	I

(1) S: Sensitive; I: Moderately sensitive; R: Resistant.

**Table 2:** Composition of in vitro fermentation medium.

Ingredient	Concentration (g/L)
Starch	8
Phospholipids	3
Chromone	3
Yeast extract powder	4.5
Bile salts	0.4
L-Cysteine hydrochloride	0.8
Hemin	0.05
NaCl	4.5
KCl	2.5
MgCl_2_·6H_2_O	0.45
CaCl_2_·6H_2_O	0.20
KH_2_PO_4_	0.40
Tween-80	1.0
Vitamin I solution ^(1)^	200

(1) Vitamin I solution: VH (biotin) 0.05 mg/L, VB_12_ 0.05 mg/L, p-aminobenzoic acid 0.15 mg/L, folic acid 0.25 mg/L, and pyridoxine 0.75 mg/L.

**Table 3:** Antibiotic sensitivity of different bovine-derived LAB fermented milks.

Antibiotic Name	Drug Content in Paper (μg/Paper)	Diameter of the Inhibition Zone (mm) (Sensitivity Category ^(1)^)		
		LpM	EhM	EfM
Kanamycin	10	33 (S)	41 (S)	31 (S)
Streptomycin	10	40 (S)	42 (S)	41 (S)
Gentamicin	10	15 (S)	13 (I)	10 (R)
Tetracyciine	30	22 (S)	21 (S)	21 (S)
Doxycycline	5	35 (S)	29 (S)	25 (S)
Erythromycin	30	31 (S)	22 (I)	17 (I)
Ampicillin	30	14 (I)	12 (R)	12 (R)
Ofloxacin	20	23 (S)	12 (R)	11 (R)
Lincomycin	15	20 (I)	20 (I)	11 (R)

(1) S: Sensitive; I: Moderately sensitive; R: Resistant.

## Data Availability

The raw 16S rRNA gene amplicon sequencing data have been deposited in the Sequence Read Archive (SRA) of the National Center for Biotechnology The corresponding BioSample accessions range from SAMN63080365 to SAMN63080384, and the SRA run accessions are SRR40621292–SRR40621311. The datasets are held under embargo until article publication.

## Abbreviations

The following abbreviations are used in this manuscript:

LAB	Lactic acid bacteria
Lp	*Lacticaseibacillus paracasei*
Eh	*Enterococcus hirae*
Ef	*Enterococcus faecalis*
LpM	*Lacticaseibacillus paracasei* fermented milk
EhM	*Enterococcus hirae* fermented milk
EfM	*Enterococcus faecalis* fermented milk
CONM	Control raw milk
GOS	Galactooligosaccharides
LOS	Lactooligosaccharides/Lactose-oligosaccharides
SCFA	Short-chain fatty acids
IgG	Immunoglobulin G
DPPH	2,2-Diphenyl-1-picrylhydrazyl
*OD*_600_	Optical density at 600 nm
RCR	Relative Change Rate
ETEC	*Enterotoxigenic Escherichia coli*
ASV	Amplicon sequence variant
OTU	Operational taxonomic unit
PCoA	Principal coordinate analysis
LEfSe	Linear discriminant analysis Effect Size
LDA	Linear discriminant analysis
MetaCyc	MetaCyc Metabolic Pathway Database
PBS	Phosphate-buffered saline
ANOVA	Analysis of variance
SAS	Statistical Analysis System
ROS	Reactive oxygen species
YCFA	Yeast extract, Casitone, and Fatty Acids medium
